# Meeting report on 14^th^ Jenner Glycobiology and Medicine Symposium: glycobiology in immunology, medicine, and clinical practice

**DOI:** 10.1093/glycob/cwac006

**Published:** 2022-02-12

**Authors:** Roisin O’Flaherty, Ghislain Opdenakker, Henrik Clausen, Rita Gerardy-Schahn, Claudine Kieda, Celso A Reis, Pauline M Rudd, Azita Sadrieh, John Axford

**Affiliations:** Department of Chemistry, Maynooth University, Maynooth, Co. Kildare, W23 F2H6, Ireland; Rega Institute for Medical Research, Department of Microbiology, Immunology and Transplantation, Herestraat 49, Leuven, KU Leuven, BE-3000, Belgium; Copenhagen Centre for Glycomics, Faculty of Health Sciences, University of Copenhagen, DK-2200 Copenhagen N, Denmark; Institute of Clinical Biochemistry, Hannover Medical School, Hannover, 30625, Hannover, Germany; Centre for Molecular Biophysics, Cell Recognition and Glycobiology, UPR4301-CNRS, rue Charles Sadron, 45071, Orléans, France; Glycobiology in Cancer, i3S – Institute for Research and Innovation in Health, University of Porto, 4200-135, Porto, Portugal; UCD School of Medicine, University College Dublin, Belfield, Dublin 4, D04 V1W8, Ireland; Bioprocessing Technology Institute, 20 Biopolis Way, #06-01 Centros, 138668, Singapore; Department of Clinical Rheumatology, St. George's University of London, London, SW17 0QT, UK; Department of Clinical Rheumatology, St. George's University of London, London, SW17 0QT, UK

**Fig. 1:**
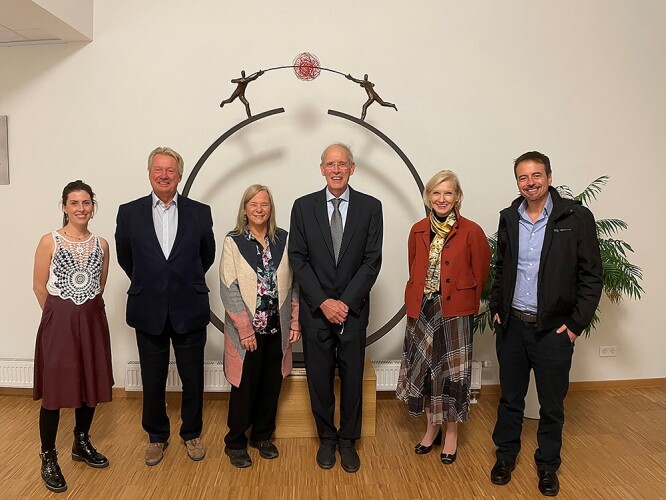
Group photo of a selection of the organizing committee at the 14^th^ Jenner Glycobiology and Medicine Symposium, October 2021, Rega Institute (KU Leuven). From left to right: Dr. Roisin O’Flaherty, Prof. John Axford, Prof. Pauline Rudd, Prof. Ghislain Opdenakker, Prof. Claudine Kieda, and Prof. Celso Reis.

The 14^th^ Jenner Glycobiology and Medicine Symposium took place at the Rega Institute, KU Leuven, in the beautiful city of Leuven, Belgium, last October 2021 (Fig. 1). The Jenner Glycobiology and Medicine symposia were the first and main international multidisciplinary conferences set up to examine the relevance of glycobiology to immunology, medicine, and clinical practice. The goal of the 14^th^ meeting was to continue to provide a free international forum for researchers to disseminate current leading-edge studies in the novel and classic fields of glycobiology, glycoimmunology, and glycomedicine. This year’s Jenner XIV Symposium was a hybrid event, with most participants and speakers attending virtually through a Zoom platform and only a small collection of in-person audience members. These comprised the chairs for the sessions and 1 invited speaker working in Belgium. Traditionally, this conference was held as an in-person event prior to this meeting. Going forward, we plan to maintain the hybrid format with in-person and online attendance to facilitate an inclusive symposium with full recording and global accessibility for all.

Across the globe, from Ireland to Brazil, a mixture of early career researchers, more established academics, and veteran investigators spoke of their cutting-edge research across 4 different sessions: “Innate and Adaptive Immunity,” “Glycosylation and Glycan Recognition,” “Translational Glycobiology,” and an interview-style session, “An interview with Sir Greg Winter.” Over 200 attendees tuned in virtually at different stages of the day. The symposium directed the audience members to major recent achievements in the field of glycoscience, and speakers described major technological advancements in fields such as atomic force microscopy and chemical editing tools (Koehler et al. 2019; O’Leary et al. 2021); novel insights into the role of glycans and binding partners in innate, and adaptive immunity, including glycosaminoglycans, glycoproteins, galectins, and *O*-glycopeptidases (Salanti et al. 2015; McCarthy et al. 2018; Madsen et al. 2020; Cagnoni et al. 2021; Pluvinage et al. 2021); and the effects of modifications, such as acetylation or sulfation (Baumann et al. 2015) and the role of glycans in Covid-19, including viral attachment and animal studies (Sanchez-Felipe et al. 2021; Harbison et al. 2022) and their clinical and translational applications (Winter et al. 1994); and the introduction to and description of a new class of molecules (glycoRNA) was described (Flynn et al. 2021). A quiet respect and homage were paid to early contributions to the field. Born through the works of Prof. Raymond Dwek (University of Oxford) and Prof. Ivan Roitt (University College London) in the 1980s, spurred on from early discoveries made in the late 19^th^ century by Prof. Emil Fischer, the field of glycoscience has since flourished and the respect and enthusiasm to those that came before were evident from all speakers.

A link to the recorded event can be found at: https://youtu.be/BFc9zSOvG7s. The next meeting—The 15^th^ Jenner Glycobiology and Medicine Symposium—will be hosted by Celso Reis at the Institute for Research and Innovation in Health, University of Porto (www.i3s.up.pt) in the city of Porto, Portugal, and we welcome students and staff with an interest in glycoscience in medicine and translational science.
